# Structural Color Painting by Rubbing Particle Powder

**DOI:** 10.1038/srep08340

**Published:** 2015-02-09

**Authors:** ChooJin Park, Kunsuk Koh, Unyong Jeong

**Affiliations:** 1 Department of Materials Science and Engineering, Yonsei University, 134 Shinchon-dong, Seoul, Korea

## Abstract

Structural colors originate from purely physical structures. Scientists have been inspired to mimic the structures found in nature, the realization of these structures still presents a great challenge. We have recently introduced unidirectional rubbing of a dry particle powder on a rubbery surface as a quick, highly reproducible means to fabricate a single crystal monolayer assembly of particles over an unlimited area. This study extends the particle-rubbing process to a novel fine-art painting, structural color painting (SCP). SCP is based on structural coloring with varying iridescence according to the crystal orientation, as controlled by the rubbing direction. This painting technique can be applied on curved surfaces, which enriches the objects to be painted and helps the painter mimic the structures found in nature. It also allows for quick fabrication of complicated particle-assembly patterns, which enables replication of paintings.

The colors of some objects in nature result from purely physical structures^1^. Such structural colors can be generated by several different optical processes, such as interference, scattering, interaction of light with 2D gratings or photonic crystals, or combinations of these processes^2^,^3^. Structural coloring shows promise in a variety of applications including sensors^4^,^5^, functional films^6^, optical filters^7^,^8^, color reflectors for display^9^, metamaterials^10^,^11^, and photonic devices^12^. To achieve structural coloring, scientists have been inspired to mimic the structures found in nature, such as the wing scales of butterflies and beetles; the spines of sea mice, which have periodic voids 500 nm apart; and the scales of tropical fish, octopus, and squid^13^,^14^. The color in these natural structures varies with spacing, tilting angle, and refractive index. Although such structures have been artificially fabricated via lithography^15^,^16^,^17^ and vacuum evaporation processes^18^, these approaches are not economical for large-area coloring. Thus, although the physical origins of structural colors are well understood, the realization of these structures still presents a great challenge.

Self-assembled spherical particles of sub-micrometer size have opened up the possibility of structural coloring based on 2D photonic crystals^19^,^20^. Given the significant advances in particle synthesis and optical modulation, the natural assembly of particles is an attractive method of structural coloring. To achieve highly-reproducible arbitrary structural coloring in a robust way, the fabrication process must meet several requirements: i) ability to repeatedly make arbitrary assembly patterns; ii) spatial control over the size and orientation of the crystal domains; iii) quick and easy structural fabrication in a scalable fashion; iv) structural uniformity on curved surfaces; and v) combination with other coloring mechanisms. To date, solution-based colloidal self-assembly has been the primary method of structural fabrication^21^,^22^,^23^,^24^,^25^,^26^. However, this approach is limited in assembly scale, and the crystal orientation is not fully controllable. In addition, the solution process is also time-consuming, and it can only achieve a uniform assembly on flat surfaces.

Recently, solvent-free particle assembly methods have been developed. Khanh *et al.* introduced the process of mechanically rubbing a particle powder on a hard template with periodic dents, which enabled precise positioning of spherical particles in the dents^27^. Michaelis *et al.* suggested edge-induced rotational shearing to generate high-quality opal thin films exhibiting tunable structural colors^20^. These dry assembly processes are quick and scalable, which is pivotal in realization of structural colors. We have recently introduced unidirectional rubbing of a dry particle powder on a rubbery surface as a quick, highly reproducible means to fabricate a single crystal monolayer assembly of particles over an unlimited area^28^. When rubbed on an elastomer surface, the particles collectively roll along the rubbing direction so that the initially separate grains merge to form a large single domain in which the particles are hexagonally assembled. This study extends the particle-rubbing process to a novel fine-art painting, structural color painting (SCP).

## Results and Discussion

**Figure 1:**
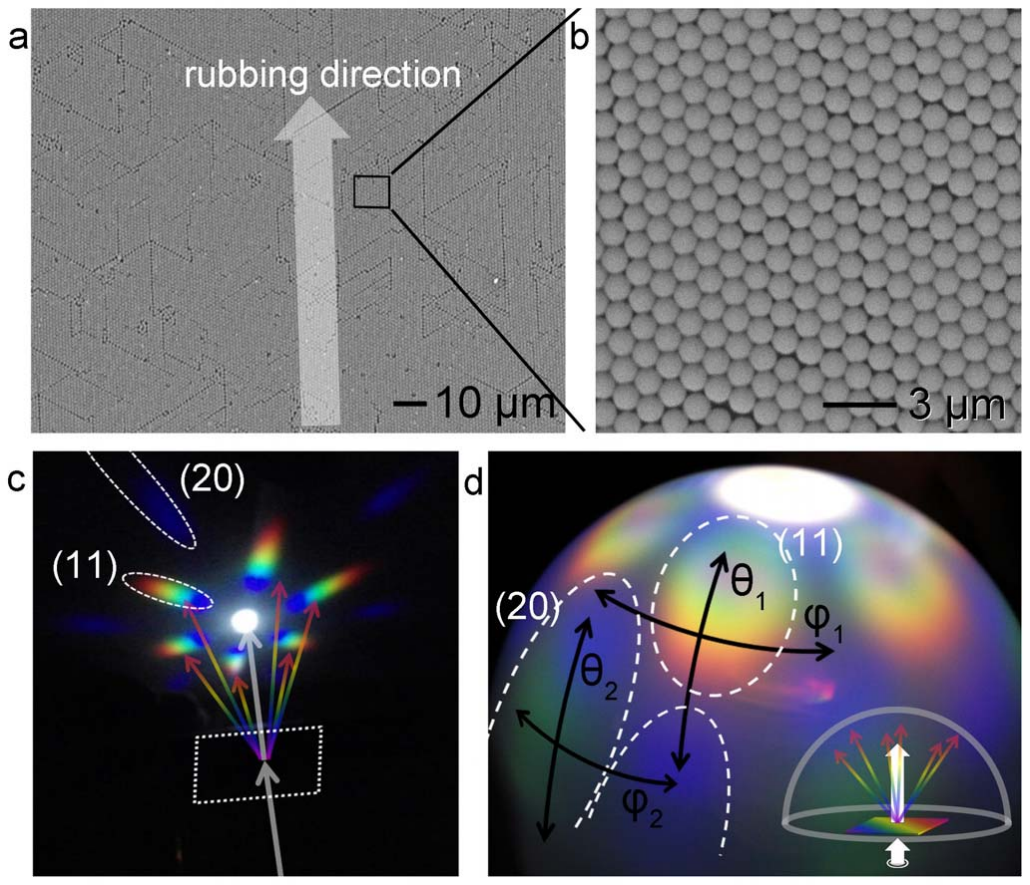
Single grain of 2D particle assembly by the rubbing process and its diffraction colors. (a) SEM image of a hexagonally-ordered polystyrene (PS) particle monolayer (1 μm in diameter). This particle monolayer was prepared by rubbing on a PDMS-coated glass slide (10 cm × 10 cm). (b) A magnified SEM image of the box in Figure 1a. (c, d) Photograph of the particle assembly diffraction on a black paper (c) and on a semitransparent half dome screen (d). White light was transmitted to the single grain monolayer perpendicularly through a hole; 1 cm for (c) and 4 cm for (d). The light diffracted from the (11) plane (20) plane was split into rainbow colors. Human eyes perceive different colors depending on the tilted angle (θ), azimuthal angle (φ), and the sample-to-eye distance.

Figure 1a shows a SEM image of an assembled particle monolayer obtained by repeatedly rubbing spherical 1-μm-diameter polystyrene (PS) particles on a 10 cm × 10 cm PDMS-coated glass plate^28^. A camra image of the rubbing process is shown in the supporting information (Figure S1). Although line defects were observed, all the particles were aligned along the rubbing direction (indicated by an arrow). The particle monolayer had uniform ordering and orientation; hence, the grains were also uniform throughout the substrate surface. Except for the line defects, the particles were well-ordered hexagonally, as seen in the higher-magnification SEM image in Figure 1b.

This 2D particle assembly diffracts light at different angles depending upon the wavelength of the incident light. At a fixed incident angle (*θ_i_*), the diffraction angle (*θ_m_*) is determined by the wavelength (*λ*) and lattice distance (*d*) between the ordered particles, as given by^2^: *d* (*n_i_* sin*θ_i_* + *n_m_* sin*θ_m_*) = *Mλ*, where *M* is the diffraction order, and *n_i_* and *n_m_* are the refractive indices of the incident and diffraction media, respectively. When the light is incident perpendicular to the flat 2D particle assembly (*θ_i_* = 0), diffraction in the air (*n_i_* = *n_m_* = = 1) yields the grating equation: 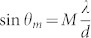. Figure 1c shows an optical image of the diffracted light on a flat white screen when white light passing through a small, 1-cm-diameter hole was irradiated perpendicularly onto the particle assembly. Since the substrate was transparent and the interface was flat, the colors shown in Figure 1c originated from diffraction by the particle assembly, not from reflections at the interfaces. The diffraction was identical in both reflection and transmission modes (Supporting information, Figure S2). The diffracted light was split into rainbow colors according to the diffraction angle (*θ_m_*), with blue at small angles, and red at large angles. Two lattices contribute to the diffraction; the diffraction from the (11) plane ranges from 32° (blue) to 48° (red), while the diffraction from the (20) plane ranges from 67° (blue) to 90° (green). Therefore, different colors appear if either the incident angle (*θ_i_*) or the viewing angle (*θ_m_*) is changed.

When the size of the beam irradiated onto the particle assembly is increased, the size of the diffraction ellipse also increases. Figure 1d shows the diffracted light on the semi-transparent hemispherical cover when white light was irradiated through a large 4-cm-diameter hole. The colors displayed on the hemisphere visualize the polar plot. In this case, the colors shown on the hemisphere changed continuously between the diffraction ellipses, resulting in a color change at the azimuthal angle (*φ*). The color change at *φ* indicates that human eyes perceive different colors when the particle assembly sample is rotated in its plane. This color change at *φ* also depends on the altitude (angle *θ*) on the screen. In Figure 1d, the color at the angle *φ*_1_ changes from orange to blue, but the color at *φ*_2_ changes from green to blue. Rotating the sample is equivalent to changing the orientation of the particle assembly, which means that the colors from two grains with different crystal orientations look different to the eye.

The optical resolution at both angles (*θ*, *φ*) is enhanced as the distance (*d*) from the sample to the hemispherical cover increases. Human eyes perceive mixed colors at a small *d* but separate colors at a large *d*^17^. When the spatial distance between two color sources is very close, human eyes cannot distinguish the colors and perceive them as one mixed color. For example, the wing scales of a *papilio palinurus* butterfly have concavities a few micrometers apart that alternately reflect blue and yellow, but human eyes interpret the concavities as green^1^,^6^. When the spatial distance between the light sources is large enough, human eyes may perceive a rainbow of structural colors. Thus, these parameters (*θ*, *ϕ, d*) make the structural colors from periodic microstructures appear to change, which was the focus of this work.

**Figure 2:**
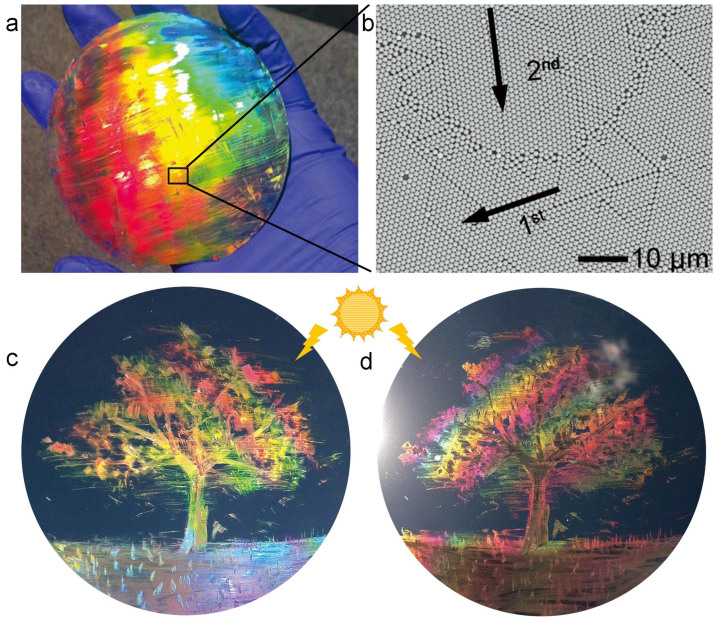
The structural coloar painting created by rubbing a powder of particles with a rubber pen. (a) Photograph of a monolayer of PS beads 1 μm in diameter on a PDMS-coated Si wafer. The monolayer was rubbed at right angles with a small piece of PDMS. The color of the monolayer changes according to the rubbing direction and the viewing angle. (b) SEM image of the area boxed in Figure 2a. The arrows indicate the rubbing directions, and the numbers indicate the rubbing sequence. The second rubbing impeded in the area receiving the first rubbing, creating a grain boundary. (c, d) A structural color painting, “A tree on a lawn on a windy day,” which was drawn by rubbing the PS beads with a rubber pen. Depending on the viewing angle, the picture appears differently in terms of both color and texture, similar to lacquer work inlaid with mother-of-pearl.

The rubbed particles were assembled along the <10> direction as an easy rolling path. Controlling the orientation of a particle assembly enables color tuning by adjusting the diffraction angle. The simple rubbing process allows for an unprecedented control of the crystal orientation and thus, the colors displayed. Figure 2a illustrates the color changes resulting from different rubbing directions. A powder of 1-µm-diameter PS particles was rubbed on a PDMS-coated Si wafer with a small piece of a 1-cm-thick PDMS stick, so that the rubbing directions intersected at right angles. A full rainbow of colors was displayed across the surface of the wafer. Figure 2b shows a SEM image taken from the boundary (the solid box in Figure 2a) between the yellow and orange areas. The crystals in the boundary region show distinct orientations and a clear crystallographic boundary. The shape of the boundary implies that the second rubbing with a rubber stick altered the large crystal monolayer created by the first rubbing. Figures 2a and 2b demonstrate the unique characteristics of the rubbing process: i) the reflected color is tunable, ii) the size of a colored area is determined by the rubbed area, and iii) a colored area can be recolored by rubbing the surface in a different direction. These color-related characteristics of the rubbing process introduce a new concept in artistic painting, termed here as “structural color painting.” Figures 2c and 2d demonstrate one such painting, “A tree on a lawn on a windy day.” It was created on a 4-inch PDMS-coated Si wafer by rubbing the 1-µm-diameter PS particles with a rubber-coated pen. Since the color of the particle assembly depends on the incident angle of the light, slightly tilting the wafer yields different color sets for the same painting, similarly to lacquer work inlaid with mother-of-pearl.

**Figure 3:**
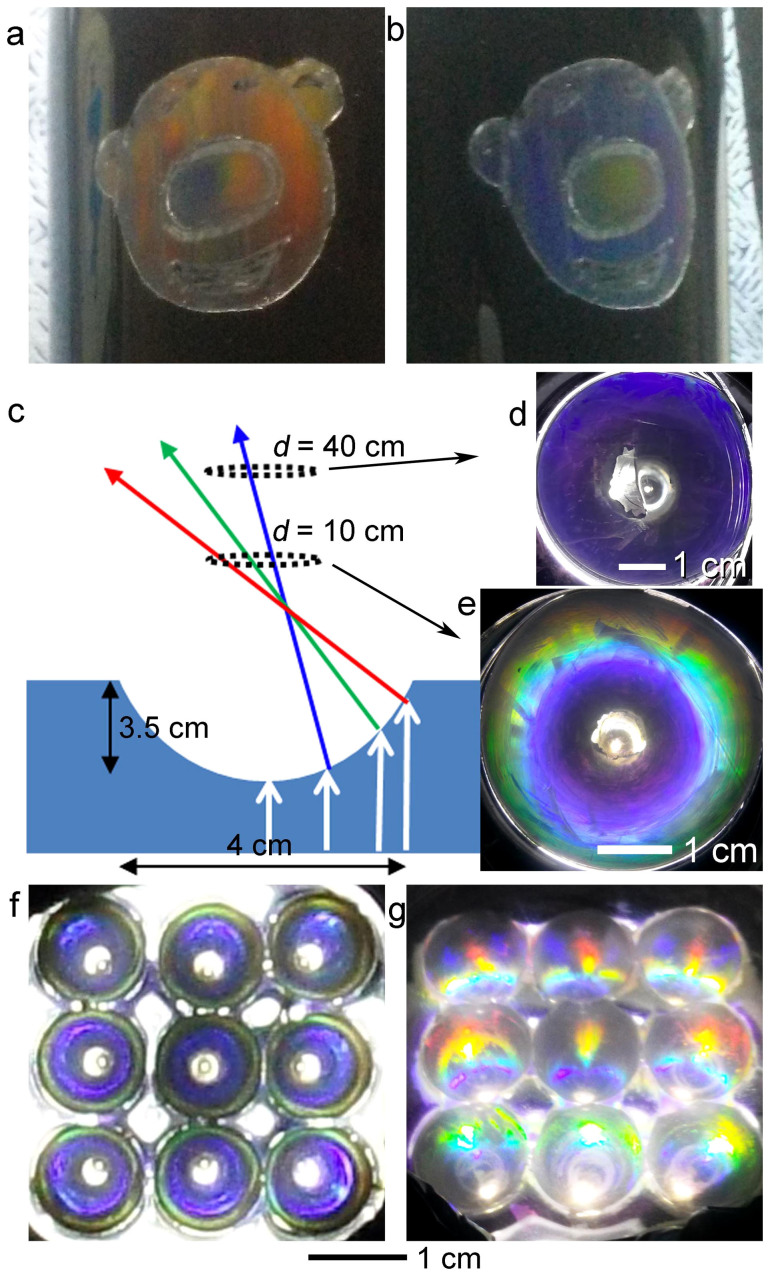
The structural color paintings on curved surfaces. (a, b) photographic images of structural color painting on a mug. On a PDMS-coated mug, a PS particle powder (1 μm in diameter) was rubbed. The colors changed according to the viewing angle. (c) A schematic for observing diffraction from a concave hemisphere with assembled particle monolayer on its surface. (d, e) Color difference depending on the viewing distance (*d*). The color looks differently; concentric rainbow at short distance at *d* = 10 cm (d), but a blue monocolor at *d* = 40 cm (e). (f) Photographic image of the diffraction colors from concave hemisphere array, shown from the front at *d* = 40 cm (f) and from tilted angle (θ = 30°).

Structural coloring based on the 2D photonic crystal painting can also be applied on curved surfaces that have rubbery characteristics. Due to good conformity between the particle monolayers and the curved surfaces, the samples displayed distinct colors depending on the viewing angle. By combining the color tuning and arbitrary painting capabilities, the use of photonic crystal painting was extended to diverse daily life objects. Figures 3a and 3b show a figure drawn by rubbing 1-μm-diameter PS particles on a PDMS-coated mug and the color changes when the mug was tilted.

Rubbing particles on a substrate with topological variations can diversify the resulting colors and designs. For example, the particle monolayer formed on the concave surface of a hemisphere displays different colors depending on the curvature of the concavity. We applied the particle rubbing process on an array of 4-cm-wide and 3.5-cm-deep hemispherical concavities fabricated on a PDMS substrate. A powder of 1.5-μm-diameter spherical silica beads was rubbed with a motorized rotating rubber ball (Supporting Information, Figure S3). The (11) plane was directed concentrically around the walls of the concavities (Supporting Information, Figure S4). Hence, the colors also varied concentrically, gradually changing from the center to the edges. When the rubbing was done in random directions, the color was not concentric (Supporting Information, Figure S5). Due to the curvature of the concavity and the particles, when white light is perpendicularly incident on the substrate from the bottom, wavelengths of 375, 450, and 488 nm are diffracted at 45, 60, and 70° curvatures, respectively (Figure 3c). The concavity looked mostly blue when viewed from a greater distance (*d* = 40 cm) (Figure 3d). When the viewing distance was small (*d* = 10 cm), different colors were perceived blue at the center, green in the middle, and yellow at the edge (Figure 3e). An array of the concavities displayed blue at *d* = 40 cm (Figure 3f). When the array sample was tilted, the concavities looked colorful (Figure 3g). The concentric colors were not observed at the tilted angles, and the structure of the colors continued changing as the tilting angle increased (Figure S6).

**Figure 4:**
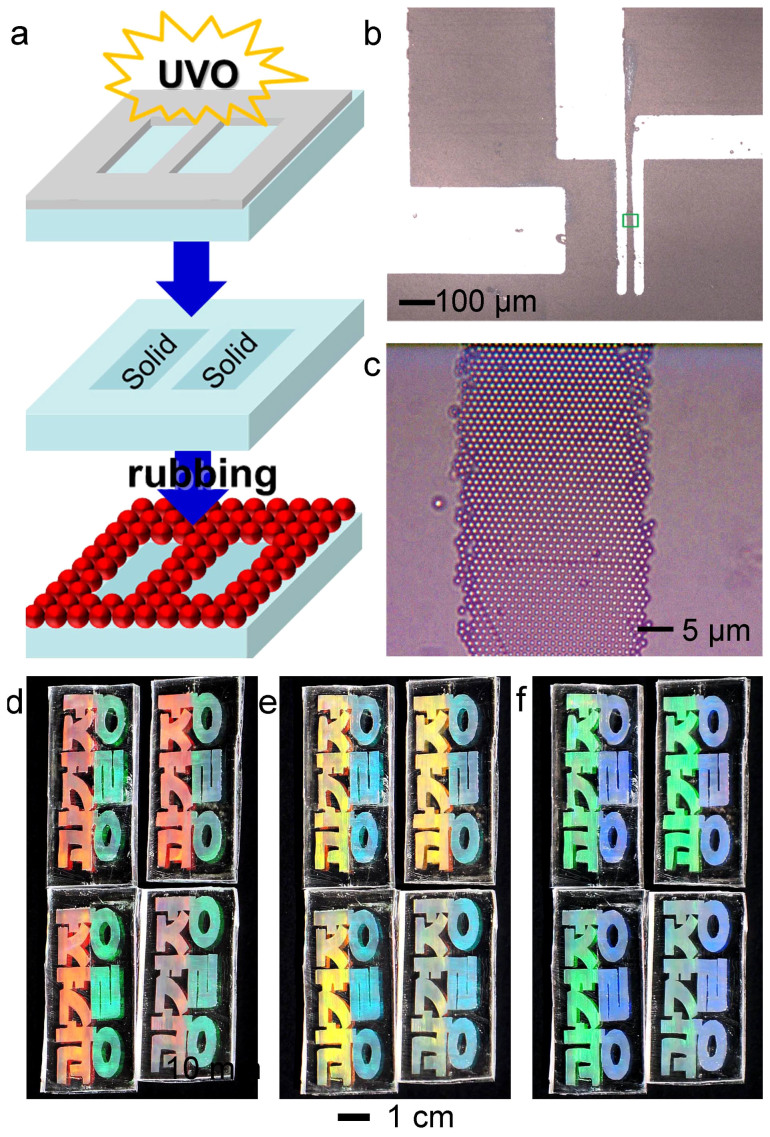
Patterning of the assembled particle monolayer. (a) Schematic of particle monolayer micro-patterning. A PDMS surface was exposed to ultraviolet ozone (UVO) through a metal mask. The exposed regions became non-sticky. The particles tended to slide from the non-sticky regions and assembled only on the sticky regions. (b,c) Micro-pattern of the particle monolayer with the same crystal orientation (b) and a magnified optical image of the box (c). (d,e,f) Replication of the structural color painting letters. The left halves were rubbed upwards and downwards. The right halves were rubbed from left to right. The colors look differently according to the viewing angles.

The monolayer assembly of micro-scale particles resulting from mechanical rubbing requires a certain level of adhesion energy (≤ 0.01 gf·mm) of the substrate. Rolling the particles enables the hexagonal packing, but if the adhesion is too low, the particle aggregates tend to slide across the substrates and therefore, are not separated into individual particles. Taking into account the minimum adhesion energy of the substrates, patterning of a colloidal monolayer could be readily achieved by modifying the surface adhesion of the substrates. Figure 4a shows the experimental procedure for the patterning of the assembled monolayer. The PDMS substrate was exposed to ultraviolet-ozone (UVO) through a metal mask before the particle powder was rubbed on the surface. The adhesion energy (*E*) of the UVO-treated surface was reduced to nearly zero (*E* = 0.001 gf·mm). As a result, the particles slipped in the non-sticky regions during rubbing and were assembled only in the neighboring sticky regions (*E* = 0.05 gf·mm)^28^. Unidirectional rubbing on the UVO-treated pattern led to uniform crystal orientation throughout the particle assembly. Figure 4b shows an example of the patterned assembly of particles (PS, 1 µm in diameter). Figure 4c is a magnified image of a 50-µm-wide line pattern. The crystal structures in the other areas had the same single domain and an identical crystal orientation (Supporting Information, Figure S7). Thus, using a mask allows for repeated production of the same assembly features. Figures 4d–f depict four structural color letters manufactured using the same mask. The left halves of the letters were rubbed upwards and downwards, whereas the right halves were rubbed from left to right. The colors of the four letters were clearly distinguished in the middle and continued changing as the tilting angle was increased.

In summary, the process of rubbing particle powder on a rubber substrate leads to monolayer assembly of the particles and allows the crystal orientation to be changed simply by changing the rubbing direction. Crystal domains with different crystal orientations display distinct structural colors when the incident light is large, as is the case in daily life. The colors could be recolored repeatedly by changing the crystal orientation. We extended this color-tuning capability to a novel type of fine art painting, structural color painting*.* This unique painting technique can be applied on both curved and flat surfaces, which allows for drawing on most everyday goods, such as the mug in this study. Rubbing particles on concave or convex surfaces enables us to mimic natural objects showing structural colors. By patterning the adhesion energy of a substrate, complicated patterns of colloidal monolayers could be fabricated.

In general, the color reflectance of a monolayer particle array is lower than the multilayer particle arrays, especially in the white background. The color reflectance depends on the target applications and on the intensity of the light. Simple monolayer gratings such as compact disks (CDs) have good color visibility and are often used in in-door or out-door decoration. The color visibility can be greatly enhanced by increasing the refractive index of the periodic features. In the particle rubbing, using high-index ceramic particles such as TiO_2_ or the organic/inorganic composites will improve the color visibility. There are several commercial products, so-called effect pigments, which display sophisticated color interplay depending on the angle of observation. Such effects are achieved on curved objects^29^. The use of spherical particles containing colorants can be another approach to diversify the monolayer structural coloring in real paintings. We expect this new painting and patterning technique can be used for lithography, diffraction masks, cosmetics, and decorations on a variety of surfaces, from small, everyday necessities to large surfaces like cars and walls.

## Methods

PDMS (Sylgard 184) was purchased from Dow Corning. The pre-polymer and cross-linker were mixed at a weight ratio of 10:1. For the rubber substrate, the mixed PDMS liquid was poured onto a polystyrene petri dish with uniform thickness (5 mm), and the cast liquid was cured at 80°C for 12 h. The wafer and glass substrates coated with PDMS were prepared by spin-coating the mixed PDMS liquid at 3000 rpm for 30 s and then cured. To pattern the adhesion energy of the rubber surface, the PDMS surfaces were exposed to ultraviolet-ozone (UVO) for 60 min. To coat PDMS on curved objects, the mixed PDMS liquid was painted with a brush and then cured. PDMS substrates with concavities were prepared by arranging nine steel balls (1 cm in diameter, 3 × 3) on a glass substrate and pouring the mixed PDMS solution, followed by thermal curing.

Any rubber that is not too sticky (E > 10 gf mm) and not too hard (E < 0.001 gf mm) can be used ref. 28. Practically, the adhesion energy of ‘too sticky’ is that of the adhesive tape, and adhesion energy of ‘too hard’ is that of a bare plastic surface. The adhesion energy of most elastic materials is in between the two regions. Therefore, the species of rubbers applicable in this process is wide enough, including PDMS rubber with a diverse crosslinker mixing ratios, block copolymer rubber film, and polyurethane rubber, etc.

To obtain the single-crystal monolayer, a dry PS powder (with particles 1 μm in diameter) was sandwiched between PDMS-coated glass substrates. The top substrate was pressed by a palm and moved unidirectionally left and right five times. For drawing large features, a long PDMS piece (1 cm × 10 cm) was cut from the PDMS thick plate (1 cm in thickness) prepared in a square petri dish (10 cm × 10 cm). For fine-art painting, the tip of a ball-point pen was coated with PDMS and used as a rubber brush. Then pen was dipped in the PDMS prepolymer solution, taken out, and heated at 70°C for 2 h in a heating chamber. The pictures were drawn by rubbing the particle powder on a PDMS-coated wafer. The pressure and shear force required to rub the particle powder does not need fine control. If the external force overcomes the adhesion between the particles and the substrate, rubbing the particles is accomplished. Readers can imagine the drawing images with a pastel, as shown in Figure S1.

For the concavity experiment, 10-mm iron bearings were arrayed with a 3 × 3 structure. Additional PDMS was poured until the thickness of PDMS reached the bearing radius. After curing, bearings were removed. A 10-mm iron bearing was held in front of an electric drill tip, and coated with PDMS. Silica powder (1.5 μm in diameter) was put in the hemispheres, and the bearing in the electric drill tip was electrically spun at 100 rpm for 10 sec. Patterned particle monolayers were fabricated by rubbing the particle powder on PDMS-coated wafers with adhesion energy patterns. The powder was covered by a large PDMS substrate and rubbed in the same way to prepare the single-crystal monolayer.

## Author Contributions

U.J. designed the experiments and guided the project. C.J.P. performed the experiments. K.S.K. contributed to particle preparation. All authors discussed the results and commented on the manuscript at all stages.
